# Molecular Detection of *Salmonella* spp. and *E. coli* non-O157:H7 in Two Halal Beef Slaughterhouses in the United States

**DOI:** 10.3390/foods12020347

**Published:** 2023-01-11

**Authors:** Omar A. Al-Mahmood, Angela M. Fraser

**Affiliations:** 1 Department of Veterinary Public Health, College of Veterinary Medicine, University of Mosul, Mosul 41002, Iraq; 2 Department of Food, Nutrition, and Packaging Sciences, Clemson University, Clemson, SC 29634, USA

**Keywords:** halal slaughterhouse, halal meat, shiga toxin-producing *E. coli*, *Salmonella* spp., virulence genes

## Abstract

The aim of this study was to estimate the prevalence of pathogenic bacteria on halal beef carcasses and environmental surfaces in two halal beef slaughterhouses in the United States. To evaluate halal beef slaughter operations, 144 beef carcass samples (pre- and post-evisceration), and 24 environmental site samples (slaughter hall floor, brisket saw, and offal’s table) were collected in two halal beef slaughterhouses during June to September 2017. All carcass and environmental samples were analyzed for the presence of *Salmonella* spp., *Escherichia coli* O157:H7, and shiga toxin-producing *E. coli* (non-O157 STEC). Results revealed that *Salmonella* spp. was isolated and confirmed for the presence of *invA* gene in 5/36 samples (13.8%) and 5/36 samples (13.8%) at pre-evisceration in plants A and B, respectively. *Salmonella* spp. was isolated in 2/9 samples (5.6%) of plants A and was not detected in any sample at post-evisceration process. *E. coli* O157:H7 was not detected in any sample collected from plant A and B. *E. coli* non-O157 was isolated and confirmed for the presence of virulence genes in 4/36 samples (11.1%) and 2/36 samples (5.5%) at post-evisceration in plants A and B, respectively. *Salmonella* spp. was detected based on the presence of the *Salmonella invA* gene in the slaughter hall floor (4/4) and the offal’s table (2/4) samples using multiplex polymerase chain reaction (mPCR). In plant B, *Salmonella* spp. was also confirmed in the slaughter hall floor (2/4) and brisket saw (2/4) samples. On the other hand, one isolate of *E. coli* O157:H7 and one non-O157 STEC were obtained from the slaughter hall floor of plant A. The *E. coli* O157:H7 isolate was positive for *stx1*, *stx2*, *eaeA*, and EHEC-*hly* genes. Two isolates of non-O157 STEC (2/4) were detected in the environmental site samples, one from the slaughter hall floor, and one from an offal’s table sample of plant B. These data can be used to inform food safety interventions targeting halal meat operations in the southeastern United States.

## 1. Introduction

One of the fastest-growing segments of the US food business, the US halal food market was valued at USD22.6 billion in 2016 and is anticipated to reach USD26.8 billion by 2021 [1]. The term “halal” which means “lawful” is used to describe food and beverages that Muslims are allowed to consume. Halal is also meant to signify high standards for hygiene and cleanliness in the food production process [2]. Halal laws are derived from the Quran and the Sunnah of the Prophet Mohammad [3]. The animal must be healthy and alive, the slaughter must be performed by a Muslim according to the prescribed rituals, and the animal’s throat must be severed with a sharp knife in one quick step to sever the carotid artery, jugular vein, and throat. The carcass should be drained of its blood sufficiently [2]. Lastly, the person who is slaughtering the animal must first make the intention of performing the slaughter then must recite an invocation, typically “Bismillah and Allahu Akbar” [4].

In the United States, as of 2018, there were 6500 meat and poultry slaughtering and processing facilities (including 89 halal slaughterhouses) [5]. The majority of halal establishments currently fall under the category of very low-volume slaughterhouses. Very low volume slaughterhouses have on average 10 employees and annually slaughter ≤ 6000 animals [6]. Not surprisingly, US halal slaughterhouses are also located in areas with large Muslim populations [5]. According to two studies, bacterial contamination is higher in extremely small-scale (non-halal) slaughterhouses than in large-scale slaughterhouses [7,8]. Smaller slaughter halls and generalized job duties performed by the same staff members at low-volume slaughterhouses increase the possibility of carcass contamination [7]. Additionally, low-volume operations frequently have less automation (more hand contact), fewer production spaces (possible increase in cross-contamination), inadequate sanitation skills, and less space overall [9]. The majority of US slaughterhouses for halal beef (more than 90%) fall under the category of very low-volume operations [5]. Low-volume operations have the same food safety requirements as large volume operations. 

Furthermore, epidemiological data has demonstrated that improperly managed animal slaughter can result in the contamination of meat with foodborne pathogens [10]. Nineteen outbreaks of foodborne illness have been linked to slaughterhouses and meat/poultry processing facilities in the US since 2006. *Salmonella* spp. was attributed to seven outbreaks in poultry processing facilities and twelve in beef processing facilities (*E. coli* O157:H7 caused nine and *Salmonella* spp. caused three outbreaks) [11]. In addition, there was a single outbreak associated with ground beef produced by Gab Halal Foods (a halal food processing facility) caused by *Salmonella enterica* Typhimurium [12].

The majority of the scientific data relevant to halal slaughter operations originates from Muslim-majority nations rather than western nations. Only two microbiological studies have been conducted on halal beef, one in a halal slaughterhouse in the US and the other in a halal butcher shop in the UK, according to a systematic literature search [5,13]. Five microbiological studies on the slaughter of beef were carried out in Middle Eastern (Islamic) nations [14,15,16,17,18], but it is challenging to make comparisons because the food safety system differs from that of developed nations.

Given how quickly the halal food market is expanding in the US, it is sensible to investigate this segment of production in order to identify potential food safety risks. Therefore, this study aims to determine the prevalence of pathogenic bacteria (*Salmonella* spp., *E. coli* O157:H7, non-O157 STEC) on halal beef carcasses and environmental surfaces in two halal beef slaughterhouses in the United States over a four-month using multiplex PCR.

## 2. Materials and Methods

### 2.1. Informed Consent

The Clemson University Institutional Review Board approved the research protocol of this study. Before data collection began at each slaughter operation, the operation manager provided written consent.

### 2.2. Sampling Frame and Sample Size 

Two halal slaughterhouses (Plants A and B) from the eight halal slaughter operations included in our geographic sampling frame agreed to participate in the study. Samples were collected from both slaughterhouses between June and September 2017. During each site visit (every four weeks for 12 visits), three carcasses were randomly selected, and samples collected during two processing steps at three carcass sites (brisket, flank, and rump), as recommended by United States Department of Agriculture-Food Safety Inspection Services (USDA-FSIS) [19]. Systematic random sampling was used to select carcasses and ensure all samples were representative of the population of animals slaughtered on the visit day. Where a starting point was randomly selected, the periodic interval was calculated by dividing the population size (N = the number of slaughtered animals per day) over the sample size (n = the number of animals that would be sampled). The total number of samples was 144 (2 slaughterhouses × 12 carcasses × 2 processing steps × 3 carcass sites). 

### 2.3. Carcass Sampling 

Swabs (100 cm^2^ each) were collected from each carcass using a carcass sampling kit (sample-Right™ dry cellulose sponge, Nasco developed Whirl-Pak^®^ bag, single-use gloves, and 25 mL Butterfield’s Phosphate Buffer (World Bioproducts LLC, Woodinville, WA, USA)) from three sites on each carcass at two slaughterhouses. A sterile template 10 × 10 cm (100 cm^2^) (World Bioproducts LLC, Woodinville, WA, USA) was used to mark the swabbing areas for the three carcass sites, which were taken (1) after hide removal-pre-evisceration and (2) at the end of slaughter after the final wash before chilling-post-evisceration. The swabbing procedure included 10 horizontal scrubbing motions followed by 10 vertical scrubbing motions for each site [20]. Each sample bag was labeled with a unique identifying code, placed in an insulated container within five minutes of data collection, then transported on ice for microbiological analysis at Clemson University. Carcass samples were processed within 12 h of collection.

### 2.4. Environmental Sampling

During the same visit to collecting carcass samples, three environmental surface sites (slaughter hall floor, brisket saw, and offal table (offal sorting and washing table)) were swabbed during slaughtering. The total of environmental samples for each slaughterhouse was 12 samples (4 visits × 3 surfaces). Surface samples were aseptically collected using sterile pre-moistened polyurethane foam PUR-Blue™ swabs by rubbing firmly over the surface area marked with a sterile template (10 × 10 cm) [21]. Each swab was labeled with a unique identifying code, placed in the shipping container within five minutes of collection, then transported on ice for microbiological analysis at Clemson University. Environmental samples were processed within 12 h of collection. 

### 2.5. Microbiological Analysis of Beef Carcasses and Environmental Samples 

All carcass and environmental samples were analyzed for the presence of *Salmonella* spp., *E. coli* O157:H7, and non-O157 STEC.

#### 2.5.1. *Salmonella* spp. Isolation and Confirmation

A total of 168 different samples (144 beef carcass samples (9 samples × 2 slaughter processes × 2 plants × 4 visit times) and 24 environmental samples) were analyzed using the ISO 6579, 2002 (updated in 2007) standard method for the detection of *Salmonella* spp. Sterile buffered peptone water (Alpha Bioscience Inc., Baltimore, MD, USA) was added to each sample at a 1:10 ratio and homogenized in a stomacher for 2 min at room temperature then incubated at 35–37 °C for 24 h. After incubation, 0.1 mL and 1 mL of pre-enriched culture were transferred to 10 mL Rappaport-Vassiliadis Broth (EMD Chemicals Inc., Darmstadt, Germany) and Tetrathionate Broth (EMD Chemicals Inc., Darmstadt, Germany) then incubated at 41.5 °C, 37 °C for 24 h, respectively. Isolates were cultured on Brilliant Green Agar (Sigma-Aldrich, St. Louis, MO, USA), Xylose Lysine Deoxycholate Agar (Difco, Sparks, NV, USA), Bismuth Sulfite Agar (Sigma-Aldrich, St. Louis, MO, USA), and Hektoen Enteric Agar (Difco, Sparks, NV, USA) then incubated at 35–37 °C for 24–48 h [22]. All presumptive *Salmonella* spp. isolates were biochemically confirmed using Triple Sugar Iron Agar (Oxoid LTD, Hampshire, UK) and Lysine Iron Agar (Oxoid LTD, Hampshire, UK).

*Salmonella* spp. isolates were confirmed using polymerase chain reaction (PCR). *Salmonella* Enteritidis (H2292) was used as a positive control. A 284 bp region of the *invA* gene was targeted and amplified for *Salmonella* spp. using 139-R (5′-GTG AAA TTA TCG CCA CGT TCG GGC AA) and 141-F (5′-TCA TCG CAC CGT CAA AGG AAC C) primers designed by [23]. Subsequently, a colony from the plate was suspended in 1 mL sterilized distilled water in a 2 mL Eppendorf tube and boiled for 10 min. Thereafter, the Eppendorf tube was chilled on ice then centrifuged at 7000 rpm for 5 min. Two microliters of the supernatant were used as template DNA in the PCR reaction.

Reactions were carried out in a total volume of 25 μL containing 2 μL of DNA template (60 ng of DNA), 1 μL (100 pmol) of each primer, 2U Taq Polymerase, 10× Taq buffer (100 mM Tris-HCl (pH8.3), 500 mM KCl), 25 mM MgCl_2_, and 2.5 mM dNTP mixture (Takara Bio Inc., Tokyo, Japan). PCR amplifications were conducted using a DNA thermocycler (Eppendorf Realplex^2^ Mastercycler, Hamburg, Germany). The PCR protocol consisted of an initial incubation for 2 min at 95 °C followed by 30 cycles of denaturing for 30 s at 95 °C, 30 s at 50 °C for annealing, and 45 s at 72 °C for extension then 7 min at 72 °C for the final extension. The PCR products were mixed with 6× loading dye and analyzed by electrophoresis on 1.2% agarose gel with TBE (Tris/Borate/EDTA) as the running buffers. Thereafter, the products were stained with ethidium bromide and visualized by UV illumination (BIO-RAD Laboratories, Milan, Italy).

#### 2.5.2. *E. coli* O157:H7 and Non-O157 STEC Isolation and Identification

A total of 168 unique samples (144 beef carcass samples (9 samples × 2 slaughter processes × 2 plants × 4 visit times) and 24 environmental samples) were analyzed for the detection of *E. coli* O157:H7 and non-O157 STEC using the ISO 16654, 2001 standard method. Samples were enriched in a modified Tryptic Soy Broth with Novobiocin (Sigma-Aldrich, St. Louis, MO, USA) at a 1:10 ratio and homogenized in a stomacher for 2 min at room temperature (Model 400, Seward Stomacher^®^, West Sussex, UK) then incubated at 41.5 °C for 18–24 h. A 1.5 mL Eppendorf tube was used for separation and concentration. One ml of the enriched broth culture was treated with 20 μL immunomagnetic beads coated with anti-O157 antibody (Dynabeads™ anti-*E. coli* O157) (Applied Biosystems, Inc., Foster City, CA, USA) for 10 min with continuous agitation using an MPC™-S rack (DYNAL Biotech, Inc., Lake Success, NY, USA) to prevent the beads from settling. Multiple washing steps using a sterile wash buffer were used to avoid cross-contamination. The Eppendorf tubes were inserted onto the Magnetic plate MPC™-L (DYNAL A.S, Oslo, Norway) for 3 min for maximum recovery of Dynabeads^®^ anti-*E. coli* O157. The sample supernatant was carefully aspirated and discarded. Dynabeads^®^-bacteria complex was resuspended in 100 μL of wash buffer and mixed briefly by vortex.

Fifty (50) μL of Dynabeads^®^-bacteria complex was inoculated onto MacConkey Sorbitol Agar containing Cefixime-Tellurite supplement (CT-SMAC) (Sigma-Aldrich, St. Louis, MO, USA) and CHROMagar™ O157 (DRG International Inc., Springfield, IL, USA) and incubated at 37 °C for 24 h. Colorless colonies on CT-SMAC and mauve colonies on CHROMagar™ O157 were examined by the indole tests (Sigma-Aldrich, St. Louis, MO, USA) and specific latex agglutination test for *E. coli* O157:H7 (Remel™ Wellcolex™, Kent, UK) to confirm the isolates before using multiplex PCR. 

In this study, a multiplex PCR reaction was performed for the detection of four gene sequences (*stx1*, *stx2*, *eaeA*, and EHEC *hlyA*) of *E. coli* O157:H7 and non-O157 shiga toxin-producing *E. coli. E. coli* O157:H7 (F6B-2) was used as a positive control. Oligonucleotide primers were manufactured commercially (Invitrogen, Life Technologies Inc., Waltham, MA, USA) (Table 1). 

*E. coli* isolates were incubated at 37 °C overnight on tryptone soya agar (Difco, Sparks, NV, USA) plates. Subsequently, a colony from the plate was suspended in 1 mL sterilized distilled water in a 2 mL Eppendorf tube and boiled for 10 min. Thereafter, the Eppendorf tube was chilled on ice and then centrifuged at 7000 rpm for 5 min. Two (2) μL supernatant was used as template DNA in the PCR reaction. 

PCR assays were carried out in a total volume 50 μL containing 2 μL DNA template (60 ng of DNA), 2 μL of 2 mM concentrations of each primer, 4 U Taq Polymerase, 10× Taq buffer (100 mM Tris-HCl (pH8.3), 500 mM KCl), 25 mM MgCl2, and 2.5 mM dNTP mixture (Takara Bio Inc., Tokyo, Japan). Temperature conditions consisted of an initial incubation for 3 min at 95 °C followed by 35 cycles of 95 °C for 20 s, 58 °C for 40 s, and 72 °C for 90 s then 5 min at 72 °C for the final extension. 

### 2.6. Statistical Analysis

Descriptive and inferential statistics were performed using JMP Pro16.1 software [27]. Percentages were calculated as a descriptive statistic. A chi-square test was used to determine whether there was a significant relationship between two slaughter processing steps and pathogenic reduction frequencies. Results were significant with a *p* < 0.05.

## 3. Results 

### 3.1. Prevalence of Pathogenic Microorganisms in Beef Carcasses

A total of 144 different samples were tested for *Salmonella* spp., *E. coli* O157:H7 and non-O157 STEC during four months of sampling (June–September 2017) of plant A and B. *Salmonella* spp. was isolated and confirmed for the presence of *invA* gene in 5/36 samples (13.8%) and 5/36 samples (13.8%) at pre-evisceration in plants A and B, respectively. 

*Salmonella* spp. was isolated in 2/9 samples (5.6%) of plants A and was not detected in any sample at post-evisceration. Table 2 shows the number of *Salmonella* spp. isolates using different media and PCR.

*E. coli* O157:H7 was not detected in any sample collected from plant A and B. *E. coli* non-O157 was isolated and confirmed for the presence of virulence genes in 4/36 samples and 2/36 samples at post-evisceration in plants A and B, respectively (Table 3).

Most interestingly, pathogenic *E. coli* (non-O157 STEC), and *Salmonella* spp. In carcass samples significantly decreased (*p* < 0.02) after the decontamination steps (final wash) in both plants. 

### 3.2. Environmental Sites Pathogenic Bacteria

A total of 24 different environmental samples were tested for *Salmonella* spp. And non-O157 STEC. *Salmonella* spp. was confirmed for the presence of *Salmonella invA* gene in the slaughter hall floor (4/4) and the offal’s table (2/4) samples using multiplex PCR. In plant B, *Salmonella* spp. was also confirmed in the slaughter hall floor (2/4) and brisket saw (2/4) samples. On the other hand, one isolate of *E. coli* O157:H7 and one non-O157 STEC were detected in the slaughter hall floor of plant A. *E. coli* O157:H7 was positive to *stx1*, *stx2*, *eaeA*, and EHEC-*hly* genes. Two isolates of non-O157 STEC (2/4) were detected in the environmental site samples, one from the slaughter hall floor, and one from offal’s table sample of plant B. 

## 4. Discussion

A limited number of studies describing halal beef carcass hygiene at slaughter are available, yet data are needed to characterize food safety risk factors. *Salmonella* spp. and *E. coli* O157 are common causes of foodborne illnesses. Evisceration and de-hiding processes can lead to contamination of carcasses during slaughter operations. Therefore, we aimed to estimate the prevalence of pathogenic bacteria (*Salmonella* spp., *E. coli* O157:H7, non-O157 STEC) on halal beef carcasses and environmental surfaces in a convenience sample of two halal beef slaughterhouses in the United States. 

In the present study, we observed a carcass contamination rate of 5.6% for *Salmonella* spp. at post-evisceration in plant A. However, there was no isolate of *Salmonella* spp. in beef carcasses at post-evisceration in plant B. The results indicated that the overall percentage of *Salmonella* spp. positive carcass samples was higher for pre-evisceration samples and decreased following the application of carcass decontamination procedures [28,29]. This suggests that decontamination interventions in plant B were more effective than plant A for reducing microbial loads. Using a high-pressure nozzle spray (i.e., automated washing cabinet-specific time for each treatment) of cold water in plant B (68 °F), hot water 180 °F, and organic acid (lactic acid 2%) has shown the effectiveness of microbial interventions at the final wash process. Our findings were similar to other published results that confirmed the efficacy of decontamination interventions at post-harvest approaches of beef slaughter [30,31].

The implementation of the food safety practices under the supervision of the USDA-FSIS showed lower microbial contamination in beef carcasses in the two halal slaughter operations (post-evisceration samples) compared to other studies, revealing higher contamination (7.1% and 7.8%) [32,33]. In addition, this study showed that the main contamination came from the de-hiding process compared to the post-evisceration process. Additionally, weather may affect beef pathogen load and has been studied previously; a study conducted in the US found a higher prevalence of *Salmonella* spp. during warmer than colder months [34], and our samples were collected in the summer. 

The incidence of non-O157 STEC was higher in pre-evisceration than post-evisceration. Additionally, the plant A showed higher non-O157 STEC contamination than plant B. The reason may be the effectiveness of automatic decontamination interventions compared to manual decontamination intervention (low-pressure to wash the carcasses (spray manually) with no set time for each treatment in plant A). Previous studies (non halal) showed that 58.3% of the beef carcasses tested in large processing plants in the United States carried at least one type of non-O157 STEC in pre-evisceration samples, however, the prevalence was reduced by using a variety of antimicrobial intervention strategies to 8.3% of the carcasses carrying non-O157 STEC at the post-evisceration process [35]. Rogerie et al. reported a lower post-evisceration non-O157 STEC prevalence (1.9%) on carcasses sampled during the summer at beef slaughterhouse in France [36]. Our findings revealed lower non-O157 STEC in beef carcasses compared to previous studies [36,37], whereas 10.7 and 11.4% of the post-evisceration samples were confirmed by multiplex PCR as carrying shiga toxin genes.

Lastly, the sanitation conditions of the slaughterhouse environment findings showed low levels pathogenic microorganisms in plant B suggesting effective implementation of sanitation standard operating procedures (SSOP) to control for environmental contamination of fecal contamination. Our review of SSOPs indicates that halal slaughterhouses implemented the routine cleaning procedures properly which can remove microbiological contamination effectively. Unlike our findings, Barros et al. found a higher contamination of *E. coli* on the floor, saw, and tables in non-halal slaughterhouse [38]. Piras et al. also found a higher contamination with *Salmonella* spp. which was isolated from 13 of 41 environmental samples (31.7%) at the end of the sampling day in non-halal slaughter activities compared with our study [39].

## 5. Limitation

The results of this study were limited to the participation of two slaughterhouses in the United States, where the other six halal slaughterhouses in our geographic sampling frame refused involvement. Therefore, our findings can only be generalized to the two sites included in the study.

## 6. Conclusions

In order to protect human health against *Salmonella* spp. and pathogenic *E. coli* infections transmissible between animals and humans, the implementation of the food safety practices (HACCP system) under the supervision of the USDA-FSIS provided significant control and prevention of microbial contamination in beef carcasses in the two halal slaughter operations in our study. Indeed, hygienic control programs, e.g., SSOP of the halal slaughterhouses, would reduce the risk of environmental contamination, which is a potential source of foodborne pathogens. The findings in this study may suggest that carcass contamination is influenced by slaughterhouse, and this could be due to variations in how the intervention is implemented. There were some sites in the slaughter environment that had varying degrees of contamination. The results of environmental samples did not show an association with carcass contamination. Our findings showed that the main contamination came from the de-hiding process compared to the post-evisceration process. Further research in non-Muslim majority countries is recommended to evaluate the microbial status of other halal animals (sheep, goat, and poultry). 

## Figures and Tables

**Table 1:** List of primer sequences, target genes, and predicted lengths of amplification products.

Target Genes	Direction	Primer Sequence	Size of PCR Amplicon (bp)	Reference
*stx1*	Forward Reverse	ACACTGGATGATCTCAGTGG CTGAATCCCCCTCCATTATG	614	[24]
*stx2*	Forward Reverse	CCATGACAACGGACAGCAGTT CCTGTCAACTGAGCAGCACTTTG	779	[25]
*eaeA*	Forward Reverse	GTGGCGAATACTGGCGAGACT CCCCATTCTTTTTCACCGTCG	890	[25]
*EHEC-hly*	Forward Reverse	ACGATGTGGTTTATTCTGGA CTTCACGTGACCATACATAT	165	[26]

**Table 2:** The occurrence of presumptive *Salmonella* spp. in beef carcasses during slaughter processes in two plants (June–September 2017).

Month	Plant	Slaughter Process	No. of Positive Samples on Different Media	XLD	BSA	HE	TSI	No. of Positive Samples (PCR)—*invA* Gene
June	Plant A	Pre	2/9	+	+	+	+	2/9
		Post	ND					ND
	Plant B	Pre	ND					ND
		Post	ND					ND
July	Plant A	Pre	1/9	+	+	+	+	1/9
		Post	1/9	+	+	+	+	1/9
	Plant B	Pre	2/9	+	+	+	+	2/9
		Post	ND					ND
August	Plant A	Pre	1/9	+	+	+	+	1/9
		Post	ND					ND
	Plant B	Pre	2/9	+	+	+	+	2/9
		Post	ND					ND
September	Plant A	Pre	1/9	+	+	+	+	1/9
		Post	1/9	+	+	+	+	1/9
	Plant B	Pre	1/9	+	+	+	+	1/9
		Post	ND					ND

ND: not detected, XLD: Xylose Lysine Deoxycholate agar, BSA: Bismuth Sulfite agar, HE: Hektoen enteric agar, TSI: Triple Sugar Iron agar.

**Table 3:** The occurrence of presumptive non-O157 STEC strains in beef carcass samples during pre- and post-evisceration in two plants using CHROMagar STEC medium and multiplex PCR.

Plant	Slaughter Process	No. of Isolates on CHROMagar STEC Medium	No. of Isolates Confirmed by Multiplex PCR	No. of Positive Gene(s)						
				*stx1*	*stx2*	*eaeA*	EHEC-*hly*	*stx1* + *eaeA*	*eaeA* + EHEC-*hly*	*stx1*+ *eaeA*+ EHEC-*hly*
Plant A	Pre-evisceration	13/36 (36.1%)	13/36	8	1	1	1	-	-	2
Plant A	Post-evisceration	4/36 (11.1%)	4/36	-	-	-	-	1	1	2
Plant B	Pre-evisceration	14/36 (38.8%)	14/36	6	5	-	1	1	-	1
Plant B	Post-evisceration	2/36 (5.5%)	2/36	1	-	1	-	-	-	-

- means genes were not detected.

## Data Availability

The article contains the original contributions discussed in the study. Further inquiries can be directed to the corresponding author.
